# *Paphiopedilum insigne* Morphological and Physiological Features during In Vitro Rooting and Ex Vitro Acclimatization Depending on the Types of Auxin and Substrate

**DOI:** 10.3390/plants10030582

**Published:** 2021-03-19

**Authors:** Monika Poniewozik, Marzena Parzymies, Paweł Szot, Katarzyna Rubinowska

**Affiliations:** 1 Subdepartment of Ornamental Plants and Dendrology, Institute of Horticultural Production, Faculty of Horticulture and Landscape Architecture, University of Life Sciences in Lublin, ul. Głęboka 28, 20-612 Lublin, Poland; monika87monika87_1987@o2.pl (M.P.); marzena.parzymies@up.lublin.pl (M.P.); 2 Department of Botany and Plant Physiology, Faculty of Environmental Biology, University of Life Sciences in Lublin, ul. Akademicka 15, 20-950 Lublin, Poland; katarzyna.rubinowska@up.lublin.pl

**Keywords:** chlorophyll fluorescence, micropropagation, slipper orchid, stress enzymes, tissue culture, water balance

## Abstract

To obtain healthy and good quality plants from in vitro cultivation, it is necessary to produce plantlets with well-developed rooting systems because they must undergo acclimatization, a final and a very difficult stage of micropropagation. In the present research, the effect of auxins NAA, IAA and IBA in concentrations of 0.5; 1; 2.5 and 5 mg·dm^−3^ on the *Paphiopedilum* *insigne* in vitro rooting was studied, and it was noted that 1 mg·dm^−3^ of IAA or IBA enabled the obtaining of a lot of rooted and good quality plantlets. The subsequent influence of the two most advantageous auxins on the acclimatization of plantlets in different substrates (sphagnum moss, sphagnum moss + substrate for orchids, substrate for orchids, substrate for orchids + acid peat) was tested, in the means of morphological features of plants and their physiological parameters, i.e., chlorophyll fluorescence (FV, Fm, Fv/Fm), stress enzyme activity (catalase, ascorbate peroxidase), and water balance. Considering all the tested features, it might be stated that the best results were obtained when explants were rooted in vitro in the presence of 1 mg·dm^−3^ of IAA and then planted ex vitro in substrate for orchids.

## 1. Introduction

*Paphiopedilum insigne* (Wall. ex Lindl.) Pfitzer (Orchidaceae), a so-called slipper orchid, is a valued pot plant that attracts orchid lovers with original flowers with lips similar to a shoe. It is also used in floral compositions as a cut flower.

The slipper orchid is typically propagated by a division of mother plants, however this method is highly unproductive [1]. There is a need to propagate identical varieties, thus generative propagation through seeds is also impossible. Therefore, plants available in trade are more and more often propagated by in vitro techniques [1,2]. Unfortunately, there are many problems in orchid tissue cultures, including *Paphiopedilum*. One of the difficult stages is a high percentage of dying plants after transferal to ex vitro conditions; therefore, it is very important to obtain good quality plantlets with properly developed roots [3]. A rooting system is necessary to allow water and nutrient intake by plants and to maintain the plants stably in soil [4]. These problems can usually be successfully solved by the use of appropriate plant hormones, mainly auxins [5].

To induce the growth and development of the rooting system, the most commonly used auxins are: 1-naphthaleneacetic acid (NAA), indole-3-acetic acid (IAA), and indole-3-butyric acid (IBA). Due to the activity of these plant hormones, root apical meristem strongly reacts by reducing the cytoplasmic zone and rapid cell differentiation. Besides, this group of growth regulators affects the rapid elongation [6], and thus limits the formation of new shoots and leaves, which was confirmed, among others, in the case of *Cymbidium forrestii* [7].

There are many reports in the literature regarding the effect of auxins on plants’ development. However, there is no information on *P. insigne* micropropagation [6]. The positive impact of NAA on the number of roots produced in tissue culture was confirmed, for example, by Asghar et al. [8] in the case of *Dendrobium nobile* ‘Emma white’, and Khatun et al. [9] on *Dendrobium* sp. IBA was proved to enhance root elongation of *Dendrobium* sp. [10], *Dendrobium primulinum* [11], *Dendrobium fimbriatum* [12] and *Dendrobium densiflorum* [13]. Mhopatra and Rout [14] showed a positive effect of IAA on the presence of *Geoderum purpureum* roots. Rahman et al. [15] showed, on an example of *Vanda tessellata*, that supplementation of the media with only one type of auxin was less effective for root induction and obtaining a large number of good quality shoots than the use of a combination of two auxins simultaneously, i.e., NAA together with IBA.

During micropropagation, cytokinins and auxins are often used together. Some publications have confirmed the good effect of NAA used together with BAP [9,16,17]. The above-mentioned plant hormones positively influenced the plant height of *Orchis catasetum* [17], the number of leaves of *Orchis catasetum* [17] and *Doritaenopsis* [16], the width of leaves of *Doritaenopsis* [16], and the length of leaves of *Dendrobium* [9]. Kabir et al. [12] confirmed the advantageous effect of NAA and IBA used for *Dendrobium* shoot elongation. A combination of cytokinins and auxins usually has a positive effect on the growth and development of shoots; however, Baker et al. [17] noted that in the case of *Orchis catasetum*, such a combination enhanced the formation of roots.

The other factor determining the growth and development of young plantlets in soil is the type of substrate. The proper soil should be characterized with a maximum water holding capacity, porosity, and permeability [18]. Michałojć and Nurzyński [19] stated that the features which should be taken into consideration while selecting the substrate are the cost of the soil, cultivated plant species, and the cost of utilization of the post-production waste. According to Zandoná et al. [20], sphagnum moss is the most commonly used substrate in the cultivation of orchids; however, the environmental changes lower the availability of the substrate, and therefore there is a need to search for alternative materials. Sphagnum moss has a large water-holding capacity and it includes growth substances. It also lowers and stabilizes pH and has antibacterial and antifungal properties, due to its acidity. Another popular substrate used in horticultural plant cultivation is peat. Peat has large water- and air-holding capacities and advantageous sorption and buffer conditions, but unfortunately, it is characterized by a low content of nutrients [21]. Moreover, according to Borowski and Nurzyński [22], peat resources are continually dwindling, which causes deteriorations in quality and increasing prices. Currently, there are special soil mixture substrates intended for orchids that contain components such as pine bark and peat. They are ideal for most of orchid species; however, their loose structure is not able to stabilize young plants upright. They contain large pieces of bark, which need composting, during which resins and phenol derivatives are excreted, which negatively affects the growth of plants. Bark, as a component of substrates for orchid cultivation, is characterized by high air capacity and low sorption capacity [21]. Production costs force orchid manufacturers to look for substrates that are an alternative to the basic substrates or components of substrates [23].

Rapidly changing conditions which appear during acclimatization to ex vitro conditions are very stressful to plants and might cause a high percentage of plantlets to die. It is possible to determine the level of stress which accompanies plants, by measuring the level of chlorophyll fluorescence. The usefulness of the method to confirm the stress occurrence induced by the environmental conditions and to monitor the growth of plantlets was confirmed in the case of *Phalaenopsis* [24]. Another factor that might be responsible for inducing stress in plant tissues is reactive forms of oxygen. To protect the plants against these conditions, the production of enzymes such as catalase and peroxidase is increased [25,26].

As a result of stress, the level of tissue saturation with water also changes, which might be checked with relative water content (RWC) and water saturation deficit (WSD). During the rooting stage, limitations of water and nutrient transfer might cause a decrease in relative water content in orchids tissues [27]. According to Diaz et al. [4], leaves formed during in vitro growth are not able to further grow in ex vitro conditions and they are replaced by new ones.

The aim of the presented work was to determine the most advantageous method to obtain rooted ex vitro plantlets of *P. insigne* from tissue culture. In the first place, the experiment was performed to choose the best composition of auxins, (NAA, IAA and IBA) used in concentrations of 0; 0.5; 1; 2.5 and 5 mg·dm^−3^ for the rooting and regeneration of *P. insigne* in vitro. Then, the subsequent effect of the two best chosen auxins, together with the type of substrate during acclimatization, were compared. Determination of the appropriate type of auxin and its concentration on rooting might enable the obtaining of a large number of *P. insigne* plantlets for proper acclimatization to produce a large amount of good quality plants ready for sale, in a relatively short time.

## 2. Results and Discussion

### 2.1. The Effect of NAA, IAA and IBA Auxins on In Vitro Rooting of Paphiopedilum insigne Plantlets

#### 2.1.1. The Morphological Features of *P. insigne* Explants during In Vitro Rooting, Depending on the Presence of Auxins in the Media

**Figure 1:**
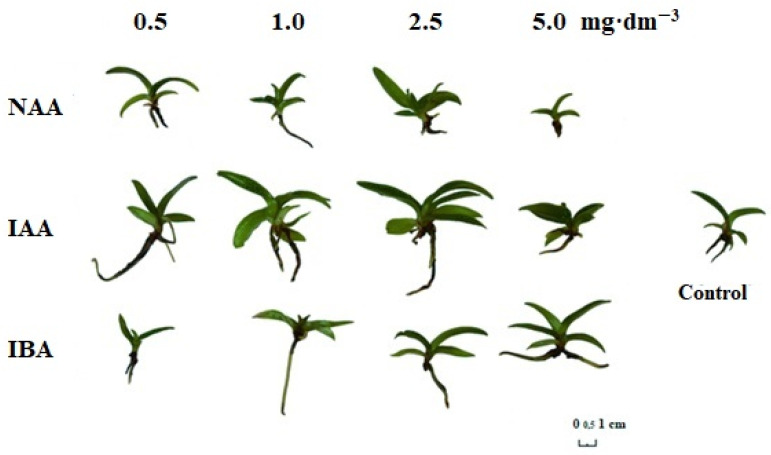
The influence of NAA, IAA and IBA auxins on in vitro rooting and morphological features of *Paphiopedilum insigne* plantlets.

Based on the obtained results, it was shown that the auxins used in the presented experiment did not significantly affect the multiplication rate of *P. insigne* explants in tissue cultures (Table 1, Figure 1).

However, comparing the results, it may be noted that the multiplication rate was the highest in the case of rosettes cultivated in the presence of 1 or 5 mg·dm^−3^ NAA (1.5), which was 50%higher than that in the presence of 1 mg·dm^−3^ IAA (1.0).

It was noted that rosettes grown on the medium without growth regulators were significantly wider (55.8 mm) than those on the medium supplemented with NAA in the concentration of 5 mg·dm^−3^ (34.5 mm) (Table 1).

The auxins used in the research affected the morphology of leaves as well. *P. insigne* plantlets with a higher number of leaves were obtained when they had been cultured on the media supplemented with 5 mg·dm^−3^ IAA, and 1 and 2.5 mg·dm^−3^ NAA (5.1; 5 and 4.5, respectively) when compared to those cultivated in the presence of NAA in the lowest and highest concentrations (3.4 and 3.8, respectively), or IBA in concentrations of 0.5 and 2.5 mg·dm^−3^ (3.8 and 3.6, respectively). The negative effect of NAA used in a concentration of 0.5 mg·dm^−3^ on the number of leaves was noted by Kishor and Sharma [28], in their experiment on the micropropagation of *Renanthera imschootiana* and *Vanda coerulea*. Different results were obtained by Talukder et al. [29] in *Dendrobium*, as well as by Jitsopakul et al. [30] in *Vanda coerulea*. The authors showed that the use of NAA in a concentration of 0.5 mg·dm^−3^ had a positive effect on the number of leaves of the tested species.

The length of leaves was also affected by the auxins used in the presented experiment. Plants with the longest leaf blades were obtained as a result of supplementation of the media with 2.5 mg·dm^−3^ IAA (29.8 mm), as compared to 1 and 5 mg·dm^−3^ NAA (23.7 and 22 mm, respectively). The number of leaves and their length determined the size of rosettes, on which the plant look depends, just as the shoot does in upright growing species. The positive effect of IAA in a concentration of 1 mg·dm^−3^ on the length of shoots was confirmed in the research of Das et al. [31] in *Cymbidium devonianum*. The negative effect of NAA applied in higher concentrations on the height of plants was confirmed by many authors, but the presented research also showed a negative influence of IBA in the lowest concentration on the studied feature, which confirms the results obtained by Baker et al. [17] in *Orchis catasetum*. Different results were obtained by Dewir et al. [3] in *Cattleya*, which formed the longest shoots in the presence of 0.6 mg·dm^−3^ of IBA.

In the presented experiment, plants characterized by the widest leaf blades were obtained on the medium supplemented with 0.5 mg·dm^−3^ NAA (13 mm) in comparison to other treatments. The positive effect of 0.5 mg·dm^−3^ NAA on the width of leaves was confirmed by Zahara et al. [32] in *Phalaenopsis* ‘Pink’.

The influence of auxins on the fresh weight of leaf rosettes was not proven in the conducted study, because the results obtained did not differ significantly.

#### 2.1.2. The *Paphiopedilum insigne* Rooting System In Vitro Depending on the Presence of Auxins in the Media

Auxins had a significant effect on the rooting of *Paphiopedilum insigne* rosettes in tissue culture (Table 2, Figure 1).

The rooting frequency of the plantlets was influenced by the auxin type and concentration. All rosettes formed roots (100%) when they were cultivated in the presence of 0.5 and 1 mg·dm^−3^ IAA or 1 and 2.5 mg·dm^−3^ IBA. Significantly fewer rosettes produced roots on the media supplemented with 0.5 mg·dm^−3^ NAA and 2.5 mg·dm^−3^ IAA (69 and 73%, respectively). Positive effects of IAA in a low concentration of 0.05 mg·dm^−3^ on the presence of roots was confirmed in the case of *Geoderum purpureum* [14], and in a concentration of 1 mg·dm^−3^ in *Dendrobium transparent* [33]. The reports of Asghar et al. [8] proved the positive influence of IBA on the tested features. The authors demonstrated the desirability of the use of IBA in concentrations of 2 mg·dm^−3^ in *Dendrobium nobile* ‘Emma White’.

The highest number of roots per explant was found in the case of *P. insigne* cultivated in the presence of 2.5 mg·dm^−3^ IAA (3.3) in comparison to those grown on the medium supplemented with 5 mg·dm^−3^ NAA (2.2). A low number of roots after supplementation of the medium with NAA was also noted by Asghar et al. [8] in *Dendrobium nobile* ‘Emma White’. Additionally, the authors stated that the increasing concentration of the auxin seedlings produced fewer roots. Reports published by Khatun et al. [9] showed that supplementation of the medium with 2 mg·dm^−3^ of NAA had a positive effect on the number of roots of *Dendrobium*, which was also confirmed in the present experiment. Different results were obtained by Parthibhan et al. [34] in *Dendrobium aqueum*. The authors reported that due to the supplementation of the medium with NAA, regardless of the concentration, they obtained the maximum number of roots.

The longest roots were observed in cases of explants cultivated in the presence of 5 mg·dm^−3^ of IAA, 0.5 and 1 mg·dm^−3^ of IBA (23.7; 23.1 and 25.1 mm, respectively) compared to those cultured on the media supplemented with 5 mg·dm^−3^ IBA and 5 mg·dm^−3^ NAA (9.8 and 9.3 mm, respectively). A positive effect of IBA in a concentration of 1 mg·dm^−3^ on the length of roots was proved in case of species such as *Dendrobium* [10] and *Dendrobium fimbriatum* [12], and in a concentration of 1.5 mg·dm^−3^ in *Dendrobium primulinum* [11] and *Dendrobium densiflorum* [13]. On the other hand, Thomas and Michael [35], noted an advantageous effect of NAA used in a concentration of 6 mg·dm^−3^ on the length of roots of *Rhynchostyilis retusa*. The influence of auxins used in the presented study on the fresh weight of the rooting system was not proved, because there were no significant differences between the treatments.

### 2.2. The Subsequent Effect of IAA or IBA and a Type of Substrate on the Acclimatization of P. insigne Plantlets to Ex Vitro Conditions

#### 2.2.1. The Survival Rate of Plantlets during the Acclimatization of *P. insigne* Depending on the Auxins used In Vitro and the Type of Substrate

**Figure 2:**
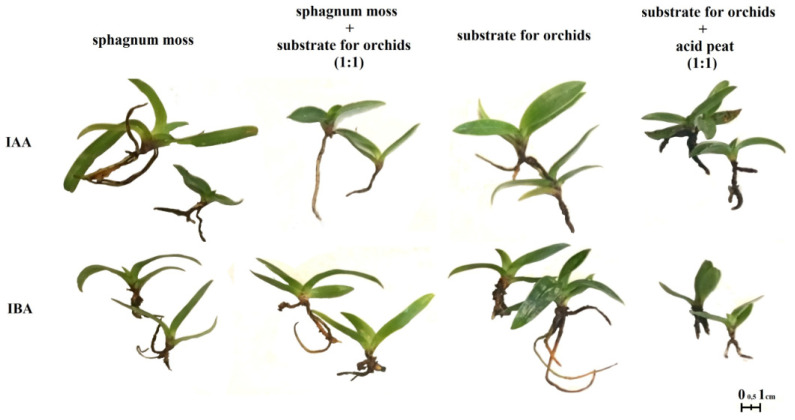
The influence of NAA, IAA and IBA auxins on in vitro rooting and morphological features of *Paphiopedilum insigne* plantlets.

The auxins used in the in vitro rooting stage influenced the growth and development of plants ex vitro (Table 3, Figure 2). It was proven that rosettes cultivated in the presence of IAA in a concentration of 1 mg·dm^−3^ in tissue culture better adapted to ex vitro conditions (55.4%), in comparison to those cultured on a medium containing IBA 1 mg·dm^−3^ (46% survival rate).

The type of substrate affected the survival rate of the plantlets during acclimatization (Table 3). It was noted that a substrate dedicated for orchids used alone or mixed with the acid peat enabled the obtaining of more adapted plants (55.8 and 51.4%) than the mixture of orchid substrate with sphagnum moss (45.1%).

Taking into consideration both factors, it was shown that the highest survival rate of plants was obtained when they were cultivated in vitro in the presence of IAA and then planted in the substrate for orchids (62%).

The obtained data do not confirm the results obtained by many authors, who proved sphagnum moss to be the best substrate for orchids. Ng et al. [36], in their study in *Paphiopedilum rotschildianum*, and Lo et al. [18] in the case of *Dendrobium tosaense*, proved the advantageous effect of sphagnum moss on the development of the studied plants.

#### 2.2.2. The Morphological Features of Rosettes during the Acclimatization of *P. insigne* Depending on the Auxins Used In Vitro and the Type of Substrate

It was also noted that the IAA or IBA used during in vitro cultivation and substrates used for the acclimatization of plantlets influenced the morphological features of the rosettes (Table 4, Figure 2).

The highest numbers of leaves were noted in the case of plantlets cultivated in vitro in the presence of 1 mg·dm^−3^ IBA and then grown in the substrate for orchids (5.1) (Table 4), which confirms the results obtained by Dewir et al. [3]. The authors showed that the use of IBA allowed the production of *Cattleya* hybrid plantlets with a definitely higher number of leaves. Fewer leaves were observed in the case of plants that were cultivated on the media supplemented with IBA and then planted into an orchid substrate and sphagnum moss mixture, or sphagnum moss alone (3.7 and 4.9, respectively). A negative influence of sphagnum moss used alone was noted in the presented experiment, which confirms the data obtained by Alves and Smozinski [37] in *Epidendrum ibaguense*, or Trelka et al. [23] in *Phalaenopsis* ‘Springfield’ and ‘Zagreb’. On the other hand, the positive effect of sphagnum moss was noted in the studies of Suradinata et al. [38] in *Dendrobium Iindii* × *Dendrobiium stratiotes* and *Dendrobium sylvanum*, as well as Juras et al. [39] in *Cattleya xanthina*. The lowest number of leaves was observed in plants that had been cultivated in vitro in the presence of IAA and then grown in the substrate for orchids (3.0). The subsequent effect of auxins alone, without a substrate, or substrate alone, without auxins, on the tested feature, was not observed.

The type of substrate, however, influenced the length and width of leaves. It was noted that plants cultivated in sphagnum moss produced longer leaves (30.8 mm) in comparison to those cultivated on sphagnum moss mixed with acid peat (26.1 mm). The widest leaf blades were noted when plantlets were cultivated in vitro in the presence of IBA and acclimatized in a mixture of sphagnum moss and substrate for orchids (8.9 mm), in comparison to those cultivated in the presence of IAA and planted into the same type of substrate (7.4 mm). The positive effect of sphagnum moss was observed by Suradinata et al. [38] in *Dendrobium Indii* × *Dendrobium stratiotes* and *Dendrobium sylvanum*, as well as by Juras et al. [39] in *Cattleya xanthina*. The negative influence of sphagnum moss on the studied feature was noted by Trelka et al. [23] in *Phalaenopsis* ‘Springield’ and ‘Zagreb’. Khatun et al. [9], during studies in *Dendrobium* hybrids, observed that the use of IBA in a concentration of 1 mg·dm^−3^, together with BAP in a concentration of 0.5 mg·dm^−3^, positively affected the width of leaves. The authors noted that the use of 2 mg·dm^−3^ IAA together with 1 mg·dm^−3^ IBA inhibited the growth of leaves in width.

#### 2.2.3. A Rooting of *P. insigne* Plantlets during Acclimatization Depending on the Auxins Used In Vitro and the Type of Substrate

The statistical analysis conducted in the presented research allows us to state that the auxins used during the in vitro rooting had a subsequent influence on the rooting of plants during acclimatization, the same as the type of substrate used (Table 5, Figure 2).

The highest number of roots was noted when *P. insigne* rosettes were cultivated in vitro in the presence of 1 mg·dm^−3^ IBA and adapted to ex vitro conditions in substrate for orchids (3.9) in comparison to other treatments. The lowest number of roots was formed when plantlets were cultivated on the media supplemented with IBA and then grown in the mixture of sphagnum moss and substrate for orchids (1.4). It was also observed that auxins used during the in vitro stage did not have a subsequent influence on root length during acclimatization, but the type of substrate did affect the length of roots. Based on the statistical analysis, it can be stated that the average length of roots was promoted by the sphagnum moss (25.5 mm) in comparison to the substrate for orchids used alone or with the acid peat (19.3 mm).

The obtained data do not confirm the results obtained by other authors who observed the negative effect of sphagnum moss on the number of roots of *Phalaenopsis* ‘Zagreb’ [23] and the length of the rooting system of *Paphiopedilum wardii* [40] and *Phalaenopsis* ‘Springfield’ and ‘Zagreb’ [23]. A substrate similar to that used in the presented research was used for the acclimatization of *Paphiopedilum insigne* in the study conducted by Deb and Jakha [41], who used a mixture of sand, decaying organic matter, brick pieces, charcoal pieces, and dried cow dung (1:1:1:1:1 *v/v* ratio); they obtained a 75% survival rate. In the protocol described by Diengdoch et al. [42], the survival rate of plantlets grown in a mixture of cocopeat + charcoal + decaying litter (1:2:1 *v/v*) was 64.9%.

### 2.3. The Physiological Response of P. insigne Plantlets during Acclimatization Depending on the Auxins Used In Vitro and the Type of Substrate

#### 2.3.1. The Influence of IAA or IBA Used In Vitro and Substrate Type on Chlorophyll Fluorescence of *P. insigne* Plantlets during Acclimatization

The auxins used during the in vitro stage and type of substrate during acclimatization influenced the physiological features of plants (Table 6).

The minimal and maximal fluorescence and the maximal quantum efficiency of PS II (Fv/Fm) were estimated in the presented experiment (Table 6). The minimal fluorescence (Fo), also called the initial because it is the first point on the chlorophyll fluorescence curve [43], was estimated in the presented experiment. The highest Fo index during the acclimatization of *P. insigne* was characterized in plants cultivated in sphagnum moss, regardless of the type of auxin (252.88), in comparison to those cultivated in the substrate for orchids (214.38). The auxins used during in vitro propagation had no influence on the fluorescence during acclimatization. However, taking into consideration both conditions applied, it was observed that plants cultivated in vitro on the medium supplemented with IBA in a concentration of 1 mg·dm^−3^ and then planted into the substrate for orchids were characterized with the lowest minimal fluorescence (182.25) in comparison to all other combinations. According to the research of Kalaji and Łoboda [44], high Fo values indicate lower efficiency of excitation energy transfer between pigment molecules in the PSII, and therefore might indicate higher stress in plants.

Maximum fluorescence (Fm) is the maximum fluorescence intensity of objects adapted to the dark [43]. In the leaves of *Paphiopedilum insigne*, it was shown that the highest Fm value was obtained when the plantlets were cultivated in vitro on the medium supplemented with 1 mg·dm^−3^ IBA and then acclimatized in the substrate for orchids mixed with acid peat (1006.00). Statistically similar results were obtained in the case of the same auxin treatment and the use of the substrate for orchids alone (982.00), and sphagnum moss mixed with soil for orchids (953.25). The lowest maximal fluorescence value was estimated when plantlets were rooted in vitro in the presence of IBA 1 mg·dm^−3^ and then planted into sphagnum moss (823.50). Considering the effect of auxins used during the in vitro rooting stage, regardless of the substrate, it was noted that the application of IBA enabled the obtaining of a significantly higher maximal fluorescence value (900.50). Taking into consideration the influence of the substrate only, regardless of the growth regulators, it was observed that the use of substrate for orchids alone or with acid peat resulted in a higher maximal fluorescence (937.63 and 958.88, respectively) in comparison to sphagnum moss used alone (870.50). According to Kalaji and Łoboda [44], a decrease in the Fm level indicates the presence of stress. The obtained results indicate that the substrate for orchids is a better substrate than sphagnum moss.

In the presented experiment, the maximum photochemical efficiency of PSII (Fv/Fm) was also evaluated. A significant decrease in the parameter value indicated damage to PSII due to present stress factors [44,45]. Unfortunately, the data given in the available literature provide diverging optimal values of the studied parameter. According to Matysiak [46], it should be between 0.75 and 0.85, while according to Johnson et al. [47] it should not be lower than 0.83. The usefulness of the studied parameter to evaluate the physiological state of plants to confirm the presence of stress was confirmed in the case of *Phalaenopsis* [24]. In the presented experiment, the highest value of the Fv/Fm ratio was found in the case of *P. insigne* plantlets rooted in vitro on the medium supplemented with 1 mg·dm^−3^ IBA and then acclimatized in the substrate for orchids or in the substrate for orchids mixed with acid peat (0.802 and 0.790, respectively). The results obtained might indicate a good condition of plants, according to the values given by Matysiak [46]. A similar indicator was given by Ebrahim et al. [48], who proved a positive effect of peat on the cultivation of strawberries. In the presented experiment, the lowest Fv/Fm value was noted when plants were cultivated in vitro in the medium supplemented with 1 mg·dm^−3^ IBA and then adapted to ex vitro conditions in sphagnum moss (0.681), which indicated the presence of stress factors and is consistent with reports from Johnson et al. [47].

The observations confirmed that sphagnum moss might be the least useful for *P. insigne* cultivation because it dried up very quickly, which is what might have put plants under stress. The influence of stress factors on the decrease in the Fv/Fm ratio has also been confirmed by Stancato et al. [27] in the case of *Cattleya forbesii* Lindl. × *Laelia tenebrosa*. De la Rosa Manzano [49] showed that, in epiphytic orchid species, the Fv/Fm ratio oscillated around 0.8 at the beginning of a drought period, and then significantly decreased. In the presented experiment, the influence of the auxins used in the in vitro stage was not proved; however, considering the effect of the substrate type on the studied feature, it was proved that the Fv/Fm value was definitely higher when plants were cultivated in the substrate for orchids (0.788) in comparison to sphagnum moss (0.708), which might indicate that the soil for orchids is a better substrate for *P. insigne* cultivation than sphagnum moss.

#### 2.3.2. The Influence of IAA or IBA Used In Vitro and Substrate Type on the Water Balance of *Paphiopedilum insigne* Plantlets during Acclimatization

The relative water content (RWC) and a water saturation deficit (WSD) in *Paphiopedilum insigne* leaves depending on the auxins used during the in vitro rooting stage and on the substrate type used during the acclimatization of plantlets were marked (Table 7).

The highest value of relative water content (RWC) was indicated in leaves of plants rooted in vitro on the medium supplemented with IBA 1 mg·dm^−3^ and then cultivated in substrate for orchids (92.4%), in comparison to those cultivated in vitro in the presence of IAA 1 mg·dm^−3^ and planted into the substrate for orchids and acid peat (86.3%), planted into sphagnum moss alone (83.6%), or in the mixture with substrate to orchids (86.3%) or cultivated in vitro in the presence of IBA 1 mg·dm^−3^ and planted into sphagnum moss (79.0%). As previously mentioned, the sphagnum moss dried up very quickly, which could expose plantlets of *Paphiopedilum insigne* to drought stress. Considering the influence of the type of substrate on the examined feature, regardless of the growth regulators, it was proved that substrate for orchids used alone was characterized with the highest RCW value (90.2%) in comparison to other substrates. It was also shown that sphagnum moss mixed with substrate for orchids or soil for orchids mixed with acid peat were better (87.1% and 86.9%, respectively) than sphagnum moss alone (81.3%). The auxins used during the in vitro rooting stage had no significant effect on the relative water content in soil. According to Stancato et al. [27], one of the reasons for the decrease in the relative water content in orchids might be drought, and the subsequent limitation of compound and water transfer to tissues.

The research conducted by de la Rosa Manzano [49] proved that the anatomical structure of the leaves might have a significant impact on the water content in tissues, which was not studied in the present experiment. The authors showed that in the grass-like, linear leaves of *Encyclia namatocaulon*, a water transport was more efficient.

The water saturation deficit (WSD) was also affected by the auxins used during in vitro rooting and the substrates used for acclimatization (Table 7).

The highest value was noted in plants cultivated in vitro in the presence of IAA or IBA and then planted into sphagnum moss (16.4% and 21.0%, respectively), in comparison to other combinations. The lowest value of WSD was obtained in the case of plants cultivated in vitro in the presence of IBA 1 mg·dm^−3^ and planted into the substrate for orchids (7.6), in comparison to IBA and the substrate for orchids and peat mixture (13.7%), or IAA and the substrate for orchids and sphagnum moss mixture (13.7%). Taking into consideration the influence of the substrate on the value of the water saturation deficit during acclimatization of the *Paphiopedilum insigne* plants, regardless of the auxins, it was noted that the obtained values corresponded to the relative water content. The highest value was obtained in leaves of plants cultivated in sphagnum moss (18.7%), while the lowest was marked in plants grown in substrate for orchids (9.8%). Sphagnum moss is the most often used substrate for orchids [20]. It is an organic substrate obtained from natural stands, which is a major disadvantage. Moreover, the studies show that during long-time cultivation its pH lowers, mainly due to the growing roots of plants, which was proven in the case of *Phalaenopsis* [50]. Another common substrate used for the cultivation of plants is peat. According to Borowski and Nurzyński [22], it positively influenced the growth of plants and decreased the WSD in the case of *Lycopersicum esculentum*. In the presented experiment, peat had no advantageous effect on the studied features.

#### 2.3.3. The Influence of IAA or IBA Used In Vitro and Substrate Type on Stress Enzyme Activity in *P. insigne* Plantlets during Acclimatization

The stress-induced enzyme levels, of catalase and ascorbate peroxidase, were also indicated in the leaves of acclimatized plants (Table 8).

Based on the statistical analysis, the influence of the auxins used during the in vitro rooting stage and type of substrate during acclimatization on the content of catalase and ascorbate peroxidase in the leaves of *Paphiopedilum insigne* was proved (Table 8). The lowest activity of catalase was found in plants rooted on the medium supplemented with 1 mg·dm^−3^ IBA and then acclimatized in sphagnum moss (0.320 U·mg^−1^ FW). Similar statistical values were noted in the case of plants rooted in vitro in the presence of IAA 1 mg·dm^−3^ and planted into the same substrate (0.480 U·mg^−1^ FW). The highest activity of catalase was marked in the leaves of plants rooted in vitro on the medium with the addition of 1 mg·dm^−3^ IBA, and planted ex vitro in the substrate for orchids (1.300 U·mg^−1^ FW), or cultivated in vitro with 1 mg·dm^−3^ IAA and planted into the mixture of soil for orchids and peat (1.287 U·mg^−1^ FW).

Taking into consideration the activity of catalase depending on the auxins used during the in vitro stage, regardless of the type of substrate, it was noted that it was significantly higher when IBA was used (0.888 U·mg^−1^ FW) in comparison to IAA (0.737 U·mg^−1^ FW). In the case of the substrates used for acclimatization, the highest activity of catalase was marked when the mixture of substrate for orchids and acid peat was used (1.127 U·mg^−1^ FW) in comparison to the substrate for orchids used alone (0.970 U·mg^−1^ FW), which was still higher than in the case of the mixture of substrate for orchids and sphagnum moss (0.753 U·mg^−1^ FW). The lowest activity of catalase was indicated in the case of sphagnum moss (0.400 U·mg^−1^ FW), and similar results were obtained in the case of Fv/Fm values.

Sofo et al. [51] reported that, as a result of stress conditions, e.g., drought or salinity, catalase activity in plant leaves is maintained, which enables the removal of H_2_O_2_. Similarly, Faisal and Anis [52] showed that plants can adapt their antioxidant enzyme defense system to alleviate the effects of oxidative stress and the activity of reactive oxygen species. Sailo et al. [25] reported that a rapid increase in the activity of catalase occurs due to an increase in ethylene production during the aging process. It is an enzyme that protects plant cells from oxidation, and with its help, reactive oxygen species are removed [26]. However, the literature reports show that changes in catalase activity are not conclusive and are dependent both on plant species tolerance [53,54] or even the part of the analyzed plant [55].

The second antioxidant enzyme, ascorbate peroxidase, was also marked. The highest activity of the enzyme studied was noted when plants were rooted in vitro in the presence of IAA 1 mg·dm^−3^ and then planted into sphagnum moss (26.087 U·mg^−1^ FW), in comparison to all other combinations. The lowest activity was obtained when the plants were rooted in the presence of the same auxin and then planted into the substrate for orchids (12.867 U·mg^−1^ FW). Relatively low activity was also obtained when the plants were cultivated in vitro in the presence of IBA and acclimatized in the mixture of substrate for orchids and sphagnum moss (0.967 U·mg^−1^ FW). Taking into consideration the auxins only, it was observed that the lowest activity of ascorbate peroxidase occurred when plants were cultivated in substrate for orchids (15.210 U·mg^−1^ FW). Significantly higher activity was obtained in the mixture of substrate for orchids with sphagnum moss (17.13 U·mg^−1^ FW) or acid peat (17.480 U·mg^−1^ FW). The highest activity was marked in the case of sphagnum moss (22.062 U·mg^−1^ FW).

Taking into consideration the auxins only, regardless of the type of substrate, it was noted that the activity of ascorbate peroxidase in leaves was significantly higher when plantlets were rooted in vitro in the presence of IAA 1 mg·dm^−3^ (18.344 U·mg^−1^ FW) in comparison to IBA 1 mg·dm^−3^ (15.210 U·mg^−1^ FW). The main role of this enzyme is to detoxify hydrogen peroxide into water. H_2_O_2_ is formed as a result of stress caused by abiotic factors, and ascorbate in its reduction process is used as an electron donor [51]. The expression of this enzyme is a plant response to environmental stress, but it also occurs during normal growth and development. This compound similarly plays a key role in regulating the levels of reactive oxygen species [56].

## 3. Material and Methods

### 3.1. The Influence of Auxins on the Paphiopedilum insigne Plantlets’ In Vitro Rooting

The starting material for the in vitro research were microplants obtained from a stabilized culture started from asymbiotic seeds germinated in vitro [57] and passaged 3 times on hormone-free media. The explants were 10–12 mm high, 4–5 mm wide, and had 3–4 completely formed leaves. The explants were placed in 300 mL Erlenmeyer flasks containing 50 mL of Murashige and Skoog medium [58] reduced by half (1/2 MS) and supplemented with thiamine (vit. B_1_)—0.05 mg·dm^−3^; pyridoxine (vit. B_6_)—0.05 mg·dm^−3^; niacine (vit. PP)—0.25 mg·dm^−3^; glycine—1 mg·dm^−3^; myo-inositol—50 mg·dm^−3^; and sucrose—15 g·dm^−3^. The media were supplemented with auxins: 1-naphthaleneacetic acid (NAA), indolyl-3-acetic acid (IAA) or indolyl-3-butyric acid (IBA) in concentrations of 0.5; 1; 2.5 or 5 mg·dm^−3^. The medium without growth regulators was treated as a control. The pH of the media was adjusted to 5.7 with 1 M NaOH and 1 M HCl before autoclaving. The media were gelled with Maxima Lab-Agar in a concentration of 6.75 g·dm^−3^ and steam-sterilized at a temperature of 121 °C and 1 MPa for 21 min.

The experiment consisted of 13 treatments with 4 repetitions containing 28 explants each. The flasks with explants were placed in a growing room at a temperature of 28 °C ± 2 °C and a 16 h photoperiod. The light source was Fluora fluorescent lights with a light intensity of 30 μmol·m^−2^·s^−1^.

The experiment lasted for 16 weeks. The studied features included multiplication rate and morphological features of the obtained plantlets: width of the leaves rosette (mm), number of leaves, length and width of leaf blades (mm), fresh weight of the leaf rosettes (mg), presence of roots (%), number and length of the roots (mm), and the fresh weight of the rooting system (mg).

### 3.2. The Subsequent Effect of Auxins and a Type of Soil on the Acclimatization of Paphiopedilum insigne Plantlets to Ex Vitro Conditions

The plant material was microplants of *Paphiopedilum insigne* derived from the same stabilized tissue culture as in the previous study. The explants were 15 mm high, 4–5 mm wide, and had 3 fully developed leaves.

The first step of the research was conducted in vitro. The explants were placed in 300 mL Erlenmeyer flasks containing 1/2 MS medium, the same as in the first experiment, and supplemented with IAA in a concentration of 1 mg·dm^−3^ or IBA in a concentration of 1 mg·dm^−3^. The auxins were chosen based on the results obtained in the first experiment, concerning the in vitro rooting of slipper orchids. The pH of the medium was adjusted to 5.7 and gelled with Maxima Lab-Agar in a concentration of 6.75 g·dm^−3^ and then steam-sterilized. The flasks with plantlets were placed in the growing room at the same conditions as in the first experiment. There were 80 explants per treatment. The in vitro rooting lasted for 12 weeks.

The obtained plantlets were transferred into soil, to ex vitro conditions. They were first washed under tap water to remove the agar residues, the bases of plantlets were dipped in the fungicide Amistar solution (azoxystrobin 250 g·dm^−3^ active substance) in a concentration of 2 mL·dm^−3^, and then the plantlets were inserted into 1 dm^−3^ plastic containers, with 5 plants per container. Different types of substrates were used: sphagnum moss, sphagnum moss + substrate for orchids (Compo Sana) 1:1 (*v/v*), substrate for orchids alone, and substrate for orchids + acid peat pH 3.4–4.5 (Hollas, Agaris Poland) 1:1 (*v/v*). The substrates’ features are presented in Table 9.

There were 4 containers with 5 plants per combination. The substrate pH was adjusted to 6.2 with agricultural lime. The containers with plants were placed in a growing room, at the same conditions as during in vitro cultivation. The containers were placed in glass tanks and covered with plastic foil to maintain the high air humidity. The plants were regularly watered. After the first two weeks, the foil was gradually removed. The research lasted for 16 weeks.

Based on the obtained results, the following features were analyzed: the survival rate (%), morphological features of leaf rosettes (number of leaves, length, and width), and the morphological features of the rooting system (number of roots, length of roots).

### 3.3. The Physiological Reaction of Plants to Stress during Acclimatization Depending on the In Vitro Pretreatment with Auxins and the Type of Substrate

The physiological features of the obtained plants were also marked.

The level of photosynthesis was estimated with the use of Fluorometer Opti-Sciences OS30p+. The analysis of the zero and maximum fluorescence (Fo and Fm, respectively) of the chlorophyll, and maximum effectiveness of the photochemical system II (PS II, Fv/Fm) were conducted on 4 leaves of plants from each treatment. The leaves were adapted to darkness for 20 min before the measurements with the use of darkening clips.

The relative water content (RWC) was assessed according to the Barrs method [59], and water deficit (WSD) according to the Stocker method [60] was also assayed. Ten disks of 9 mm diameter were taken with a cork bore from the leaves of randomly selected plants. They were weighed on an analytical balance weight with an accuracy of three decimal places (m1). Then, they were immersed in distilled water for 24 h and placed in a shaded place. After this time, the discs were blotted dry and reweighed (m2). The discs were dried at 105 °C and their dry weight was determined by re-weighing (m3). WSD and RWC indexes were calculated according to the following formulas: RWC = (W*c*/Ws)·100%; WSD = [(Ws − W*c*)/Ws]·100%, where W*c* is the current water content in the tissue: W*c* = m1 − m3, and Ws is the water content in tissues when they are fully saturated with water: Ws = m2 − m3.

The activity of the catalase and ascorbate peroxidase were also determined. Leaves (0.2 g) were homogenized in a mortar in 0.05 mol·dm^−3^ phosphorus buffer, pH 7.0, comprising polyvinylpyrrolidone (PVP) and ethylenediaminetetraacetic acid (EDTA) at 4 °C. The homogenate was then centrifuged at 10,000× *g* for 10 min at 4 °C. The supernatant thus obtained was used for further procedures. The activity of the ascorbate peroxidase was marked according to the Nakano and Asada method [61]. The reactive mixture included 1.8 mL of phosphorus buffer, pH 6.0, 100 µmol of enzymatic extract, 20 µmol 5 mM solution of sodium ascorbinian, and 100 µL of hydrogen peroxide. The measurement of the absorbance decrease was conducted between the first and fifth minute from the reaction start, at the 290 nm wavelength. The activity of the ascorbate peroxidase was calculated with the use of the millimolar absorbance coefficient, which was 2.8. The activity of catalase was determined with the use of the Chance and Meahly [62] method, modified by Wiloch et al. [63]. The reaction mixture included 2.9 cm^3^ of 15 mM H_2_O_2_ in 50 mM phosphorus buffer, pH 7.0, and 0.1 cm^3^ of enzymatic extract. The extinction was measured for 3 min, with the use of a spectrophotometer, reading the initial and final values. The measurement was performed at the 240 nm wavelength. The activity of the catalase was calculated as an amount of H_2_O_2_ dissolution, wherein one unit of activity stands for the dissolution of 1 µmol of H_2_O_2_ over one minute. The decrease in absorbance expressed in ΔE/min by 0.0055 corresponds to 1.25 units of catalase activity.

### 3.4. Statistical Analysis

The obtained data were analyzed statistically with a Statistica 13.1 software (StatSoft), according to one-way or two-way ANOVA for one- or two-factorial design. The significance between the means was estimated with Tukey’s confidence intervals at the 5% level of significance.

## 4. Conclusions

The auxins NAA, IAA and IBA influence morphological features of *P. insigne* rosette and root tissue cultures. The best quality rosettes, in terms of the means of their height and length of leaves, were obtained on the media supplemented with 1 mg·dm^−3^ of IAA, while the number of leaves was the highest in the presence of 5 mg·dm^−3^ IAA.

Media supplemented with 1 mg·dm^−3^ IBA enabled the obtaining of 100% of rooted plants in vitro with the largest number and longest roots. However, taking into consideration both the morphological features of seedlings and the rooting, IAA in a concentration of 1 mg·dm^−3^ was proved to be the most advantageous auxin to be used in the rooting of *P. insigne* explants. The highest number of the obtained rooted plants was noted as a result of medium supplementation with 1 mg·dm^−3^ IAA and acclimatization in the substrate for orchids.

Regarding the physiological features of plants during acclimatization, it might be concluded that substrate for orchids, eventually supplemented with peat, is the most advantageous method to adapt plants to ex vitro conditions.

Taking into consideration all the tested features, it might be stated that both auxins IAA or IBA, at a concentration of 1 mg·dm^−3^, might be used to root plantlets in vitro, which then should be acclimated in the substrate for orchids alone, or eventually, with the additions of peat.

## Figures and Tables

**Table 1:** The influence of NAA, IAA and IBA auxins on the multiplication rate and morphological features of *Paphiopedilum insigne* rosettes and leaves in tissue culture.

Treatment		Mn ^1^ Rate (pcs)	Width of Rosettes (mm)	Number of Leaves per Rosette	Length of Leaves (mm)	Width of Leaves (mm)	Weight of Leaf Rosettes (mg)
Auxin (mg·dm^−3^)							
Control	0	1.3 a ^2^	55.8 a	4.3 ab	28.0 ab	9.2 b	496.4 a
NAA	0.5	1.1 a	44.3 ab	3.4 b	26.6 a–c	13.0 a	399.9 a
	1	1.5 a	40.5 ab	5.0 a	23.7 bc	8.1 b	375.1 a
	2.5	1.2 a	42.4 ab	4.5 a	26.4 a–c	8.9 b	463.6 a
	5	1.5 a	34.5 b	3.8 b	22.0 c	7.2 b	439.0 a
IAA	0.5	1.2 a	44.5 ab	4.3 ab	28.1 ab	8.3 b	451.1 a
	1	1.0 a	45.3 ab	3.9 ab	28.1 ab	8.4 b	475.8 a
	2.5	1.2 a	45.4 ab	4.6 ab	29.8 a	8.7 b	553.8 a
	5	1.3 a	43.2 ab	5.1 a	27.5ab	8.3 b	480.0 a
IBA	0.5	1.2 a	39.9 ab	3.8 b	24.4 a–c	9.6 b	407.1 a
	1	1.2 a	44.2 ab	4.1 ab	27.9 ab	9.3 b	475.5 a
	2.5	1.1 a	44.2 ab	3.6 b	25.7 a–c	9.2 b	456.9 a
	5	1.2 a	37.9 ab	4.3 ab	25.7 a–c	9.1 b	420.7 a

^1^ Mn rate, multiplication rate; ^2^ means followed by the same letter in the columns do not differ significantly at *α* = 0.05.

**Table 2:** The influence of NAA, IAA and IBA auxins on the rooting of *Paphiopedilum insigne* plantlets in tissue culture.

Treatment		Rooting Frequency (%)	Number of Roots/Explant	Length of Roots (mm)	Weight of Roots (mg)
Auxin	Concentration (mg·dm^−3^)				
Control	0	94 ab ^1^	2.9 ab	20.8 ab	179.8 a
NAA	0.5	69 c	2.6 ab	21.5 ab	214.3 a
	1	91 ab	2.5 ab	19.8 ab	170.5 a
	2.5	84 a–c	2.9 ab	18.3 ab	208.1 a
	5	82 a–c	2.2 b	9.3 c	113.5 a
IAA	0.5	100 a	2.6 ab	17.9 ab	147.7 a
	1	100 a	2.3 ab	16.3 a–c	295.6 a
	2.5	73 bc	3.3 a	21.0ab	144.4 a
	5	89 a–c	2.9 ab	23.7 a	220.3 a
IBA	0.5	82 a–c	2.6 ab	23.1 a	228.0 a
	1	100 a	2.4 ab	25.1 a	208.6 a
	2.5	100 a	2.7 ab	18.5 ab	156.3 a
	5	94 ab	2.3 ab	9.8 bc	128.7 a

^1^ means followed by the same letter in the columns do not differ significantly at α = 0.05.

**Table 3:** A subsequent effect of 1 mg·dm^−3^ IAA and 1 mg·dm^−3^ IBA auxins and substrate type on the survival rate of *Paphiopedilum insigne* plantlets during ex vitro acclimatization (%).

Type of Substrate	Type of Auxin (1mg·dm^−3^)		Mean for Substrate
	IAA	IBA	
sphagnum moss	53.7 ab ^1^	47.3 b	50.5 AB
sphagnum moss + substrate for orchids (1:1)	56.8 ab	33.3 c	45.1 B
substrate for orchids	62.0 a	49.6 b	55.8 A
substrate for orchids + acid peat (1:1)	49.0 b	53.7 ab	51.4 A
Mean for auxins	55.4 A	46.0 B	

^1^ means followed by the same letter do not differ significantly at α = 0.05.

**Table 4:** The subsequent effect of IAA or IBA and substrate type on the morphological features of the *P. insigne* plantlets during acclimatization.

Auxin (1 mg·dm^−3^)	Type of Substrate	Number of Leaves	Mean for Substrate	Length of Leaves (mm)	Mean for Substrate	Width of Leaves (mm)	Mean for Substrate
IAA	sphagnum moss	4.8 ab ^1^	4.3 A	30.3 a	30.8 A	7.9 ab	8.0 A
IBA		3.7 cd		31.3 a		8.1 ab	
IAA	sphagnum moss + substrate for orchids (1:1)	4.6 a–c	4.3 A	24.2 a	26.1 B	7.4 b	8.1 A
IBA		4.9 bc		28.1 a		8.9 a	
IAA	substrate for orchids	3.0 d	4.3 A	28.2 a	26. 8 AB	7.9 ab	8.2 A
IBA		5.1 a		25.3 a		8.4 ab	
IAA	substrate for orchids + acid peat (1:1)	4.3 a–c	4.0 A	28.8 a	29.7 AB	8.0 ab	8.2 A
IBA		4.2 a–c		30.6 a		8.3 ab	
Mean for auxins	IAA	4.2 A		27.9 A		8.1 A	
	IBA	4.0 A		28.8 A		7.8 A	

^1^ means followed by the same letter do not differ significantly at α = 0.05.

**Table 5:** A subsequent effect of IAA or IBA and substrate type on the rooting of *P. insigne* during acclimatization.

Auxin (1 mg·dm^−3^)	Type of Substrate	Number of Roots/Plant	Mean for Substrate	Length of Roots (mm)	Mean for Substrate
IAA	sphagnum moss	2.9 bc ^1^	2.8 A	25.2 a	25.5 A
IBA		2.7 bc		25.7 a	
IAA	sphagnum moss + substrate for orchids (1:1)	1.4 d	2.3 A	24.8 a	23.1 AB
IBA		3.1 b		22.0 a	
IAA	substrate for orchids	2.9 bc	3.4 A	17.0 a	19.3 B
IBA		3.9 a		21.7 a	
IAA	substrate for orchids + acid peat (1:1)	2.7 bc	2.6 A	16.2 a	19.3 B
IBA		2.4 c		22.4 a	
Mean for auxins	IAA	2.8 A		20.6 A	
	IBA	2.5 A		23.0 A	

^1^ means followed by the same letter do not differ significantly at α = 0.05.

**Table 6:** A subsequent effect of IAA or IBA and substrate type on minimal fluorescence (Fo), maximal fluorescence (Fm) and maximal quantum efficiency of PS II (Fv/Fm) in leaves of *Paphiopedilum insigne* during acclimatization.

Auxin (1 mg·dm^−3^)	Type of Substrate	Fo	Mean for Substrate	Fm	Mean for Substrate	Fv/Fm	Mean for Substrate
IAA	sphagnum moss	243.75 a ^1^	252.88 A	917.50 bc	870.50 B	0.735 a–c	0.708 B
IBA		262.00 a		823.50 d		0.681 c	
IAA	sphagnum moss + substrate for orchids (1:1)	213.75 ab	228.12 AB	879.50 cd	916.38 AB	0.755 a–c	0.750 AB
IBA		242.50 a		953.25 a–c		0.743 a–c	
IAA	substrate for orchids	246.50 a	214.38 B	893.25 cd	937.63 A	0.773 ab	0.788 A
IBA		182.25 b		982.00 ab		0.802 a	
IAA	substrate for orchids + acid peat (1:1)	260.75 a	236.00 AB	911.75 bc	958.88 A	0.707 bc	0.748 AB
IBA		211.25 ab		1006.00 a		0.790 a	
Mean for auxins	IAA	241.19 A		900.50 B		0.742 A	
	IBA	224.50 A		941.19 A		0.754 A	

^1^ means followed by the same letter do not differ significantly at α = 0.05.

**Table 7:** A subsequent effect of IAA or IBA used in vitro and substrate type on relative water content (RWC) and water saturation deficit (WSD) in leaves of *Paphiopedilum insigne.*

Auxin (1 mg·dm^−3^)	Type of Substrate	RWC (%)	Mean for Substrate	WSD (%)	Mean for Substrate
IAA	sphagnum moss	83.6 bc ^1^	81.3 C	16.4 ab	18.7 A
IBA		79.0 c		21.0 a	
IAA	sphagnum moss + substrate for orchids (1:1)	86.3 b	87.1 B	13.7 b	13.0 B
IBA		87.8 ab		12.3 bc	
IAA	substrate for orchids	88.1 ab	90.2 A	11.9 bc	9.8 C
IBA		92.4 a		7.6 c	
IAA	substrate for orchids + acid peat (1:1)	86.3 b	86.9 B	12.4 bc	13.1 B
IBA		87.6 ab		13.7 b	
Mean for auxins	IAA	86.1 A		13.6 A	
	IBA	86.7 A		13.7 A	

^1^ means followed by the same letter do not differ significantly at α = 0.05.

**Table 8:** A subsequent effect of IAA or IBA and substrate type on the catalase and ascorbate peroxidase content in *P. insigne* leaves during acclimatization.

Auxin (1 mg·dm^−3^)	Type of Substrate	Catalase (U mg^−1^ FW)	Mean for Substrate	Ascorbate Peroxidase (U mg^−1^ FW)	Mean for Substrate
IAA	sphagnum moss	0.480 cd ^1^	0.400 D	26.087 a	22.062 A
IBA		0.320 d		18.037 b	
IAA	sphagnum moss + substrate for orchids (1:1)	0.540 c	0.753 C	18.877 b	17.13 B
IBA		0.967 b		15.150 d	
IAA	substrate for orchids	0.640 c	0.970 B	12.867 e	15.210 C
IBA		1.300 a		17.553 bc	
IAA	substrate for orchids + acid peat (1:1)	1.287 a	1.127 A	15.547 b–d	17.480 B
IBA		0.967 b		19.413 b	
Mean for auxins	IAA	0.737 B		18.344 A	
	IBA	0.888 A		15.210 B	

^1^ means followed by the same letter do not differ significantly at α = 0.05.

**Table 9:** Features of the substrates used during the acclimatization of *Paphiopedilum insigne* plantlets to ex vitro conditions.

**Substrate for Orchids**	
COMPO SANA	Ready-to-use substrate for all species of orchids. Prepared on the basis of peat, contains all necessary nutrients and pine bark. Contents: peat (decomposition H_3_–H_5_), pine bark, calcium, NPK fertilizer, pH 5.0–6.5.
Sphagnum moss	Prepared from various species of sphagnum moss. It is the least decomposed peat with plants still visible. Decomposes slowly so that there is no risk of too high a level of nitrogen. It is very porous (82–85%) with high water-holding capacity. pH 3.0–4.5.
Acid peat	Fine decomposed, fine texture, lower pore space and air-filled poor space than sphagnum moss. A total pore space of around 80%. pH 3.5–4.5.

## Data Availability

All the required data which is relevant to the presented study, are included in the manuscript.
